# Anatomical analysis of the left upper lobe of lung on three-dimensional images with focusing the branching pattern of the subsegmental veins

**DOI:** 10.1186/s13019-020-01323-8

**Published:** 2020-09-29

**Authors:** Tamami Isaka, Shota Mitsuboshi, Hideyuki Maeda, Takuma Kikkawa, Kunihiro Oyama, Masahide Murasugi, Masato Kanzaki, Takamasa Onuki

**Affiliations:** Department of Thoracic Surgery, Tokyo Women’s Medical University, 8-1 Kawada-cho, Shinjuku-ku, Tokyo, 162-8666 Japan

**Keywords:** Pulmonary segmentectomy, Three-dimensional imaging, Pulmonary vein, Vessels, Lung cancer

## Abstract

**Background:**

A clear understanding of the anatomical characteristics of the pulmonary veins (PVs) is essential for the successful performance of segmentectomy and important to avoid intraoperative pulmonary vessels injury. However, there is no report showing the relations between the branching patterns of PVs and pulmonary arteries (PAs). Moreover, internationally accepted symbols for describing PVs remain unavailable. For anatomically assessing the branches and courses of the subsegmental veins in the left upper lobe (LUL), the diverse branching patterns of blood vessels and bronchi should be investigated.

**Methods:**

The branching patterns and intersegmental courses of PVs were assessed by performing three-dimensional image analysis of the bronchi, and PAs and PVs in the LUL in 103 patients who were scheduled to receive segmentectomy in LUL from January 2008 through August 2012.

**Results:**

Branching types of the bronchi and pulmonary vessels failed to be independent each other. Although the combinations of anterior extension type of bronchus with the inter-lobar type (IL-type) of arterial branching pattern were often observed, but those with the mediastinal type (M-type) were rarely observed. The combinations of apical vein dominant type with the IL-type of arteries, and intermediate and central vein types with the M-type were often observed. Since LUL was adjoined by various subsegments, and the intersegmental pulmonary veins showed diverse patterns.

**Conclusions:**

This study found the relationship among PA, PV, and bronchus patterns, in the subsegment where the branching patterns were fixed in 103 cases. This study discovered PVs that was difficult to be named by the conventional naming systems because of the diversity of the locations in the subsegment.

## Background

Pulmonary segmentectomy is developed as a surgical procedure for treating infection such as tuberculosis and bronchiectasis arising primarily in the bronchi. Recently, this procedure is used for treating small peripheral lung cancers, metastatic lung tumors, benign lung tumors, and inflammatory diseases [1–6]. While the main indication of surgery is switched from bronchial lesion to peripheral lesion, the image processing capability of computed tomography (CT) markedly is improved, and techniques for three-dimensional (3D) CT imaging are developed. For enhancing the safety and facilitating the performance of thoracoscopic segmentectomy associated with a limited visual field, surgical process can be simulated by employing 3D images preoperatively [6–13]. Although the courses of subsegmental veins between segments and subsegments are an important factor in segmentectomy [14], subsegments are known to have considerable variations in size, shape, and adjoining subsegments among patients. During surgery, it is often difficult to designate the subsegmental pulmonary vein (PV) on the basis of conventional nomenclature. In addition, no clear relations among the various branching patterns of pulmonary vessels and bronchi are reported. As previous studies reported, an intraoperative vessels injury was more frequent during the left upper lobectomy [15–17], this study anatomically analyzed the branches and courses of subsegmental PVs, which are characterized by considerable diversity in the branching patterns of blood vessels and bronchi, in the left upper lobe (LUL) of lung.

## Patients and methods

One hundred-three patients (60 men and 43 women), who were scheduled to undergo surgeries for treating lesions in LUL of lung from January 2008 to August 2012, were enrolled in this study. The mean age was 66.2 years (range: 22 to 89 years). The underlying diseases were primary lung cancers in 66 patients (64.0%), metastatic lung tumors in 18 (17.5%), inflammatory diseases in 14 (13.6%), and benign pulmonary tumors in 5 (4.9%), which were atypical adenomatous hyperplasia in 2, pulmonary hamartoma in 2, and pulmonary sclerosing pneumocytoma in 1.

**Fig. 1:**
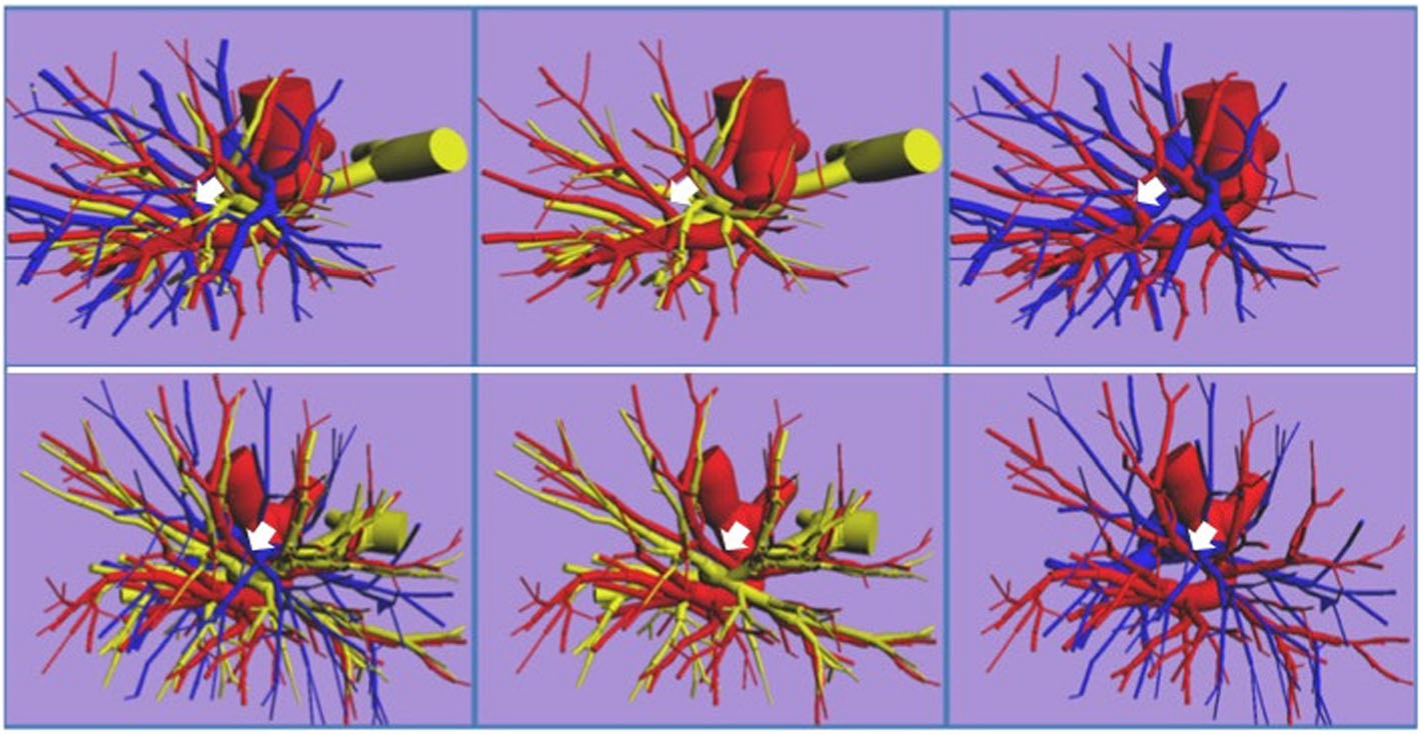
Typical 3-dimensional reconstructed images of the left lung prepared from 1-mm high-resolution computed tomography slices. Blue tubes indicate the pulmonary veins; red, the pulmonary arteries; yellow, bronchi. The image can be rotated by 360 degrees freely, allowing the pulmonary arteries and veins, and the bronchi to be reproduced, moved, and erased. These models can be used to simulate surgery. The upper row shows a case in which the lingular pulmonary arteries are running from the pars interlobaris, and the pulmonary vein branching patterns is the apical vein dominant type. The lower row shows a case in which the lingular pulmonary arteries are running from pars mediastinalis, and the pulmonary vein branching patterns is central vein dominant type. The left columns show the bronchi, and pulmonary arteries and veins; the middle column, the pulmonary arteries and bronchi; the right column the pulmonary arteries and veins

Three-dimensional images were constructed from CT images as described previously [6, 8, 10, 11, 13]. Before surgery, plain chest CT slices with a thickness of 1 mm were obtained, and high-resolution CT (HRCT) images were converted into DICOM data, which were input into a personal computer. Three-dimensional images were reproduced by surface rendering with the use of CTTRY (homemade software) and 3D modeling shareware “Metasequoia” (http://metaseq.net/en/index.html) (Fig. 1). The branches patterns of the bronchi, PAs, and PVs were assessed on the prepared 3D images. Practically, PVs were removed from the 3D-images, as the first step, the branching bronchi appeared on the images were named with concerning the branches of bronchi mainly and the branches of PVs slightly by referring to Yamashita’s classification [18], and as the second step, the segments and subsegments were identified. Naming the branching bronchi and identifying the segments and subsegments were performed by two thoracic surgeons, and for difficult cases in naming the items, other surgeons were asked to join to the naming processes.

Branching patterns of the bronchi, PAs, PVs were classified according to Yamashita’s classification [18]. Therefore, the bronchial branching patterns were sorted into three types; apico-anterior extension type, subapico-anterior extension type, and anterior extension type, by the location of anterior segment in LUL (Table 1). PA branching types were divided into two types; inter-lobar type (IL-type), in which all arteries are running into the lingular segment from pars interlobaris, and mediastinal type (M-type), in which lingular arteries are running wholly or partly from pars mediastinalis. PV branching patterns were sorted into three types; apical vein dominant, central vein dominant, and intermediate types (Table 2). The combinations of branching type of PAs and bronchi, and of PAs and PVs were studied. For PVs, the numbers of adjoining subsegments and subsegments were determined and compared by referring the nomenclature systems of Boyden [19, 20], Yamashita [18], Arai [21], and Nomori and Okada [22].

**Table 1:** The numbers of patients with the various combinations of branching patterns of the pulmonary arteries and the bronchi according to Yamashita’s classification [18] in the left upper lobes (LUL)

	Bronchial type			
	**Apico-**	**Subapico-**	**Anterior**	
	**anterior**	**anterior**	**extension type**	
	**extension type**	**extension type**		
Pulmonary arteries				Total
IL-type	17 (16.5%)	31 (30.1%)	18 (17.5%) ^a,^*	66 (64.1%)
M-type	16 (15.5%)	18 (17.5%)	3 (2.9%) ^b,^*	37 (35.9%)
Total	33 (32.0%)	49 (47.6%)	21 (20.4%)	103

The bronchial branching patterns were sorted three types; apico-anterior extension, subapico-anterior extension, and anterior extension types. The letter “1 + 2” in the illustrations means the apicoposterior segment, the letter “3” anterior segment and the letter “4” upper lingual segment of LUL. M- and IL-types indicate LUL having lingular pulmonary artery all or partly from pars mediastinalis, and lingular pulmonary artery all from pars inter lobaris, respectively. The numbers having superscripts ^“a”^ and ^“b”^ are significantly higher and lower than the theoretical numbers, respectively. *, *p* < 0.05 (Chi-square test)

**Table 2:** The numbers of patients with the various combinations of branching patterns of the pulmonary arteries and veins according to Yamashita’s classification [18]

	Pulmonary vein type			
	Apical vein dominant type	Intermediate type	Central vein dominant type	
Pulmonary arteries				Total
IL-type	51 (49.5%) ^a,^*	15 (14.6%) ^b,^*	0 ^b,^*	66 (64.1%)
M-type	18 (17.5%) ^b,^*	16 (15.5%) ^a,^*	3 (2.9%) ^a,^*	37 (35.9%)
Total	69 (67.0%)	31 (30.1%)	3 (2.9%)	103 (100%)

The veinous branching patterns were sorted three type; apical vein dominant, intermediate, and central vein dominant types. The letter “AV” means apical vein, “CV” central vein and “LV” lingular vein. M- and IL-types indicate LUL having lingular pulmonary artery all or partly from pars mediastinalis, and lingular pulmonary artery all from pars inter lobaris, respectively. The numbers having superscripts ^“a”^ and ^“b”^ are significantly higher and lower than the theoretical numbers, respectively. *, *p <* 0.05 (Chi-square test)

For determining the number of branches of the subsegmental PVs, as shown in Table 3, a matrix consisting of all subsegments in LUL in both x- and y-axes was prepared, and assuming a possibility that every subsegments were able to face to other subsegments, PVs were re-displayed with bronchi on a computer screen, the number of the branches of PVs in the subsegments and between the subsegments were counted without prejudgment, and the numbers were written in the matrix.

**Table 3:** The numbers of branches of subsegmental pulmonary veins found to course within subsegments or between subsegments in 103 patients and a comparison of the names of the pulmonary veins according to the nomenclature systems proposed by Boyden [19, 20], Yamashita [18], Arai [21], and Nomori and Okada [22]

Sub-segment	S^1 + 2^a	S^1 + 2^b	S^1 + 2^c	S^3^a	S^3^b	S^3^c	S^4^a	S^4^b	S^5^a	S^5^b	LLL	Total
V^1^l				55								55
				Y								
S^1 + 2^a	38	92	17	6	8	83	2	0	0	0		246
	B,Y	B,Y,A,N			B	Y,A,N						
S^1 + 2^b		21	92	3	0	2	0	1	0	0		119
			B,Y,A,N									
S^1 + 2^c			17	41	1	1	57	2	1	0	3	123
				B,A,N								
S^3^a				4	39	8	48	14	2	0	2	117
					Y,N		B,A	B				
S^3^b					11	85	16	66	24	0		202
					B	Y,A,N	N	Y,A				
S^3^c						14	1	3	1	0		19
S^4^a							5	46	37	7	3	98
								Y,N	Y,A	B		
S^4^b								22	73	9	1	105
									B,Y,A,N	B		
S^5^a									6	52	0	58
										Y,A,N		
S^5^b										3	1	4
Total	38	113	126	109	59	193	129	154	144	71	10	1146

V^1^l: l means lateral and was separately counted, because this subsegmental pulmonary vein courses between the region involving S^1 + 2^ a, b, and c, and S^3^a. The sites of segmental veins are marked with the nomenclature systems proposed by Boyden (B), Yamashita (Y), Arai (A), and Nomori and Okada (N). The abbreviation “S^1 + 2^” indicates the apicoposterior segment; “a”, apical; “b” subapical posterior; “c”, horizontal. “S^3^” indicates the anterior segment; “a”, lateral; “b”, medial; “c”, superior. “S^4^” indicates the lingular superior segment; “a”, lateral; “b”, anterior. “S^5^” indicates the lingular; “a”, superior; “b”, inferior. LLL indicates the left lower lobe

For statistical analysis, Chi-square tests (two sided) were used to evaluate the significance of dependencies between the groups.

This study was approved by the institutional ethics committee of Tokyo Women’s Medical University (No. 2760). Patients had both oral and written information regarding the procedure.

## Results

### Distributions of branching patterns of the bronchi, PAs, and PVs according to Yamashita’s classification

 (1) Branching patterns of the bronchi.

In 103 patients, apico-anterior extension type was found in 33 patients (32.0%); subapico-anterior extension type, in 49 patients (47.6%); anterior extension type, in 21 patients (20.3%) (Table 1).

(2) Branching patterns of PAs.

IL- and M-types were found in 66 (64.1%) and 37 (35.9%) patients, respectively (Tables 1 and 2).

(3) Branching patterns of PVs.

Apical vein type was found in 69 patients (67.0%); intermediate type, in 31 patients (30.1%); central vein type, in 3 patients (2.9%) (Table 2).

### Combinations of branching patterns

1. Combinations of branching patterns of the bronchi and PAs are shown in Table 1. There were significant dependencies (*p* < 0.05) on the chi-square test, suggesting that the branching patterns of the bronchi and of PAs were dependent. Combinations of anterior extension type with the IL type of PAs were frequently observed.
2. Combinations of the branching patterns of PAs and PVs were significantly dependent (*p <* 0.05) (Table 2). The combinations of apical vein type with IL-type of arteries, and intermediate and central vein types with the M type were frequently observed.

### Number of branches of the subsegmental PVs

The total number of branches of the subsegmental PVs in 103 patients was 1145, and the mean number of branches of the subsegmental veins per patient was 11.1 ± 1.8 (range: 7 to 16). The total number of PVs running between subsegments was 1005 with a mean number per patient of 9.8 ± 1.5 (range: 6 to 14). The total number of PVs running within subsegments was 140 with a mean number per patient of 1.4 ± 1.1 (range: 0 to 4). Among the subsegmental PVs, the number of branches arising from the superior branch of the left PV was 466 (46.4%), and the number of branches arising from the middle branch of the left superior PVs was 539 (53.6%). Table 3 shows the comparison of the numbers of branches of PVs according to the nomenclature systems of Boyden [19, 20], Yamashita [18], Arai [21], and Nomori and Okada [22]. Although many subsegmental PVs were tended to be found among subsegments as defined by these investigators, a relatively large number of other subsegmental PVs were also found.

## Discussion

Video-assisted thoracic surgery (VATS) has been performed to many pleuro-pulmonary diseases, especially early stage lung cancer. As the branching pattern of pulmonary vessels is diverse, dissecting around the arterial trunk of in LUL are intertwined to bronchi and veins, resulting in a potential risk of uncontrollable intraoperative bleeding. As the left upper lobe lobectomy has been considered as the most challenging procedure technically, previous reports show that conventional VATS shows a catastrophic rate of 1 to 1.5% and pulmonary arterial bleeding was more frequent during VATS left upper lobectomy [15–17]. It is necessary to clarify the relationship between the various branching patterns of pulmonary vessels and bronchi.

In this study, the central vein type was found in only 3 patients (2.9%), which was definitely lower than the frequency reported by Yamashita (17.5%) [18] (Table 2). Arai also reports that the central vein type is found in 2% patients [21]. Therefore, the value of 3% in this study was considered correct. The frequencies of other branching types were almost the same as reported Yamashita.

There was a significant correlation between the combinations of pulmonary arterial branching patterns and bronchial branching patterns. The combination of PA pattern also correlated with that of PV branching pattern. No previous study has reported that the relations between the branching patterns of PAs and that of bronchi nor veins. Combinations of anterior extension type of bronchi with the IL-type were often observed, while those with M-type arterial branching pattern were rarely observed. Recently, Onuki et al. have reported a possibility of the axis rotation of LUL during the embryogenic period [23]. In their report, they hypothesize that in a case having M-type arterial branching pattern, the axis of a bronchial branch, which is supposed to become the apicoposterior bronchus in IL-type, rotates forward and a bronchial branch, which is supposed to become the anterior bronchus, becomes a part of lingular bronchi. Their hypothesis indicates that the anterior bronchus has a difficulty in becoming the anterior extension type with requiring the large axis rotation of LUL, and in this study, a significantly lower number of cases with the anterior extension type bronchi was found in M-type of arterial branching pattern, supporting those of the report of Onuki et al. [23].

Boyden first uses the letters of the alphabet to simply name the subsegments [19]. Because the subsegmental bronchi and arteries also run through the subsegments, the names of these structures are designated by the same letter of the alphabet used to name the subsegment concerned. However, running between subsegments, many PVs are unable to be designated by the same letters of the alphabet used to name the subsegments, similar to the bronchi and the PAs. Although Yamashita prepares a textbook in which expressions are similar close to the nomenclature of Boyden [19], he supports the proposal that intersegmental PVs running between S^2^ and S^3^ should be designated as V^2/3^. Therefore, he names PVs running subsegments by using the name of the adjacent subsegment such as “V^1^a, v apicalis between S^1^a and S^1^b.” Arai [21], and Nomori and Okada [22] use definitions similar to the nomenclature of the subsegmental PVs. However, the distribution of subsegments is extremely diverse, and the subsegmental PVs are unable to be completely designated based on only currently available nomenclature. In fact, by comparing the number of subsegmental PVs named by the aforementioned investigators with the numbers of PVs mutually adjoining to subsegments, many branches of PVs that never named by the investigators were found to exist, whereas observing PVs adjoining to subsegments on reconstructed 3D images prepared from CT image data of 103 patients, this study was unable to name those PVs, because the veins were uncommon (Table 3).

When the difference between the results of previous studies and this study is considered, upon naming PVs in their individual cases, the previous studies name PVs after the patters of branching PVs with ignoring the definitions between the subsegments in some cases, and in other words, the previous studies are speculated to name PVs independently for adjusting the practically reasonable shapes of subsegments of which shapes are known to be highly diverse. In this study, however, searching the suitable names for PVs was easily performed with the help of 3D images prepared from HRCT image data, but precise investigations on the shapes and configurations of the bronchi, PAs, and PVs were found to be impossible in the bronchus and vessel moldings used in the previous studies. Therefore, the difference between the results of previous studies and this study was speculated to be originated from the difference in observed objects; moldings in the previous studies and 3D images reconstructed by PC in this study.

Upon using moldings in the previous studies, the bronchi and the surrounding area near PVs should be removed for inspecting PVs, and the previous studies were speculated to be unable to investigate the bronchi, PAs, and PVs more precisely. For example, although the relationship among the branching patterns of PAs, bronchi, and PVs shown in Tables 1 and 2 was one of the important factors for the anatomy of pulmonary segmentation in terms of not only orientating segmentectomy but also investigating the structural formations of pulmonary segmentation, there is no report showing the branching patterns of PAs, bronchi, and PVs.

As possible important limitations, this study was a retrospective single institutional study with a small sample size. Especially, for the subsegment naming process, the authors used their own decisions, and there was a possibility that other more clearly procedures might be developed. The results in this study need to be confirmed in a multicenter study with a larger number of patients.

## Conclusions

This study mainly investigated the anatomy of PVs in LUL and found the relationships among the patterns of PAs, the bronchi, and PVs. At a subsegment where mainly the branching bronchi were found in individual cases, upon naming PVs, this study found PVs that was difficult to be named by conventional naming methods due to the diversity of the configuration between the subsegments and PVs.

## Data Availability

The datasets used are available from the corresponding author on reasonable request.

## Abbreviations

PV: Pulmonary vein
PA: Pulmonary artery
LUL: Left upper lobe
IL: Inter-lobar
M: Mediastinal
CT: Computed tomography
3D: Three-dimensional
HRCT: High-resolution computed tomography
DICOM: Digital Imaging and Communications in Medicine
VATS: Video-assisted thoracic surgery
S: Segment
V: Vein
